# Higher Education in Times of COVID-19: University Students’ Basic Need Satisfaction, Self-Regulated Learning, and Well-Being

**DOI:** 10.1177/23328584211003164

**Published:** 2021-03-15

**Authors:** Julia Holzer, Marko Lüftenegger, Selma Korlat, Elisabeth Pelikan, Katariina Salmela-Aro, Christiane Spiel, Barbara Schober

**Affiliations:** University of Vienna; University of Helsinki; University of Vienna

**Keywords:** COVID-19, higher education, self-determination theory, well-being

## Abstract

In the wake of COVID-19, university students have experienced fundamental changes of their learning and their lives as a whole. The present research identifies psychological characteristics associated with students’ well-being in this situation. We investigated relations of basic psychological need satisfaction (experienced competence, autonomy, and relatedness) with positive emotion and intrinsic learning motivation, considering self-regulated learning as a moderator. Self-reports were collected from 6,071 students in Austria (Study 1) and 1,653 students in Finland (Study 2). Structural equation modeling revealed competence as the strongest predictor for positive emotion. Intrinsic learning motivation was predicted by competence and autonomy in both countries and by relatedness in Finland. Moderation effects of self-regulated learning were inconsistent, but main effects on intrinsic learning motivation were identified. Surprisingly, relatedness exerted only a minor effect on positive emotion. The results inform strategies to promote students’ well-being through distance learning, mitigating the negative effects of the situation.

To contain the spread of the COVID-19 pandemic, many countries instituted temporal closures of higher education institutions in March 2020. According to UNESCO (2020a), by the end of April 2020, schools and higher education institutions were closed in 178 countries, affecting roughly 1.3 billion learners worldwide. As a consequence, students have been facing a fundamentally altered situation not only with respect to their studies but also with their lives as a whole, due to manifold containment measures. Lockdowns, restrictions on movement, disruption of routines, physical distancing, curtailment of social interactions, and deprivation of traditional learning methods have led to increased stress, anxiety, and mental health concerns for learners worldwide (UNESCO, 2020b). On the whole, COVID-19 and its containment measures have created unique challenges for psychological well-being. To counteract negative developmental outcomes, resources must be identified that foster resilience in times of crisis. Therefore, the present research seeks to identify resources that support psychological well-being of students in higher education institutions in this unprecedented situation.

## Self-Determination Theory as a Framework for Resilience

Resilience, the capacity to overcome hardships, to flourish in the face of challenges (Ryff & Singer, 2003), and to activate resources, including taking chances to experience feelings of well-being (Ungar, 2005), has consistently been associated with basic psychological need satisfaction (e.g., González et al., 2019; Riggenbach et al., 2019; D. A. Thomas & Woodside, 2011; Trigueros et al., 2019). Accordingly, self-determination theory (SDT; Deci & Ryan, 2000) states that the basic psychological needs for competence, autonomy, and relatedness represent core conditions for personal growth, integration, social development, and psychological well-being. These theoretical assumptions have been consistently proven empirically across different life domains and samples (e.g., Amorose & Anderson-Butcher, 2007; Reinboth & Duda, 2006; Riggenbach et al., 2019; Van den Broeck et al., 2016). Moreover, basic psychological need satisfaction can act as a buffer in times of stress, reducing appraisals of stress and promoting adaptive coping (Vansteenkiste & Ryan, 2013; Weinstein & Ryan, 2011). The need for competence refers to experiencing one’s behavior as effective. For example, students feel competent when they are able to meet the requirements of their studies. The need for autonomy refers to experiencing one’s behavior as volitional and self-endorsed. For instance, students feel autonomous when they willingly devote time and effort to their studies. Finally, the need for relatedness refers to feeling connected with and experiencing mutual support from significant others (Deci & Ryan, 2000, 2008b; Niemiec & Ryan, 2009). In order to enable personal growth, intrinsic motivation and psychological well-being, basic psychological need satisfaction has been increasingly taken up and promoted in the educational context in recent years, with SDT acting as a framework for interventions (e.g., Guay et al., 2008; Lüftenegger et al., 2016; Reeve, 2002; Reeve & Jang, 2006).

## Distance Education and Basic Psychological Need Satisfaction in Times of COVID-19

Beyond the economic return to individuals and to society as a whole (e.g., Baum et al., 2010), higher education has the potential to improve quality of life in various ways. The role of higher education institutions in the European Union is not only to impart knowledge but also to develop the whole student by providing opportunities for personal growth and thriving. In order to enable students to become successful, resilient members of society, universities convey a range of transversal skills such as complex and autonomous thinking, creativity, and effective communication (European Commission, 2017). Moreover, universities are social spaces, enabling social interaction, offering the chance to build networks and new friendships, and generating a sense of identity and belonging with regard to the institution (Tonon, 2020). Concluding, universities represent an important developmental context for students to develop and unfold their potentials and to experience sense of belonging. The temporal closures of universities due to COVID-19 therefore represent an unprecedented challenge for students’ quality of life and thriving.

As an emergency response to the pandemic, universities worldwide have switched to distance education, marked by a rapid transition of face-to-face classes to online learning systems (Marinoni et al., 2020; Murphy, 2020). *Distance education*, or *distance learning*, is understood as an umbrella term, as its implementation varies greatly from case to case. A general characteristic is, however, the lack of physical presence and the lesser extent of informal discourse and spontaneous interaction. This bears the risk of transactional distance, a communication gap that creates negative emotions, gaps in understanding, and misconceptions (Moore, 1993). To counteract, it is crucial to explicitly address learners’ individual needs, feelings, and difficulties in distance learning environments (e.g., Richardson et al., 2015). Also, in light of numerous studies that report associations between social relatedness and academic success in both traditional and distance learning settings, interaction among learners in any learning setting should be explicitly supported (Giesbers et al., 2014; Heublein et al., 2017; Smith & Naylor, 2001; Tomás-Miquel et al., 2016). Moreover, there is consistent evidence that relatedness contributes to psychological well-being (e.g., Connell et al., 2012; Olsson et al., 2013; Reis et al., 2000; Weich et al., 2011). This further highlights the relevance of maintaining social contacts during the COVID-19 pandemic, whether with fellow students in a distance learning setting or with significant others from out-of-university contexts.

In addition to promoting social relatedness in virtual learning groups, distance education bears the potential to also promote experienced competence and autonomy when providing learners with opportunities to practice and apply what they are learning at their own pace (Paechter & Maier, 2010). In this way, students are challenged according to their abilities, and can test and expand them autonomously. Research has shown that individualized, autonomous learning environments create optimal conditions for learners to experience themselves as competent (Niemiec & Ryan, 2009). Both autonomy and competence are necessary conditions for intrinsic motivation, according to SDT (Ryan & Deci, 2000).

With all the advantages of individualized learning opportunities, it must be taken into account that self-directed and individual learning requires the learner to deal with flexibility. Learners have to structure and organize their learning themselves to a greater extent, and are required to independently integrate their learning into everyday life. Accordingly, academic success in distance education settings has repeatedly been associated with self-regulated learning competences (e.g., Geduld, 2016; Toaldo Avila & Bragagnolo Frison, 2015; Vanslambrouck et al., 2019). It has been shown, that the application of self-regulatory strategies within web-based instructions can improve learners’ self-efficacy and motivation (Chang, 2005). Moreover, in self-directed learning settings, self-regulated learning fosters students’ sense of control, thus increasing positive emotions (Pekrun, 2006). The role of self-regulated learning should therefore not be neglected when it comes to investigating distance education in times of COVID-19.

## The Present Research

To support students’ psychological well-being in times of COVID-19, it is necessary to identify resources of well-being in the current, unprecedented situation. In this respect, SDT (Deci & Ryan, 2000) represents a promising framework. Recognizing the educational context as an important setting that provides opportunities for personal growth and thriving, the present research examines to what extent basic psychological need satisfaction acts as a buffer for university students’ psychological well-being during the COVID-19 pandemic. We thus investigate whether basic need satisfaction of competence and autonomy with respect to one’s studies and experienced relatedness with significant others relate to psychological well-being. Following Deci and Ryan (2008a), we understand well-being as consisting of both hedonic and eudaimonic well-being. We therefore include positive emotion and psychological functioning, that is, intrinsic learning motivation, to operationalize students’ psychological well-being. Based on SDT, we expect that all three basic needs, namely, competence, autonomy, and relatedness, predict psychological well-being in terms of positive emotion and psychological functioning (i.e., intrinsic learning motivation; Hypotheses 1a and 1b). However, existing studies report that experiencing competence and autonomy in a distance learning setting is related to self-regulated learning (e.g., Geduld, 2016; Toaldo Avila & Bragagnolo Frison, 2015; Vanslambrouck et al., 2019). Thus, we assume that the relations between experienced competence and positive emotion and between experienced autonomy and positive emotion will be moderated by self-regulated learning (Hypotheses 2a and 2b). Accordingly, we hypothesize that the relations between experienced competence and intrinsic learning motivation and between experienced autonomy and intrinsic learning motivation will be moderated by self-regulated learning (Hypotheses 2c and 2d).

Comprising data from Austria (Study 1) and Finland (Study 2), the present research takes a multistudy approach. To examine whether findings are consistent in both countries, we first collected data in Austria and then conducted a follow-up in Finland.

## Method

### Participants and Procedure

The overall sample comprised 7,724 university students (28.3% males, 71.0% females, 0.7% diverse) with a mean age of 25.76 years (*SD* = 7.52, *Mdn* = 23.00, range = 18–71). Data were collected via online questionnaires in spring 2020. Before being forwarded to the items, participants were informed about the study’s goals; approximate duration of the questionnaire; inclusion criteria for participation, that is, attending university in the respective country; and the complete anonymity of their data. All students participated voluntarily and only those who gave active consent were included in the dataset.

#### Study 1: Austria

The sample comprised 6,071 university students (30.7% males, 68.9% females, 0.4% diverse) with a mean age of 25.02 years (*SD* = 6.90, *Mdn* = 23.00, range = 18–71). The students were from higher education institutions all over Austria. Data were collected from April 7 to April 24. We distributed the link to the online questionnaire by contacting diverse stakeholders such as university rectorates and higher educational networks. Additionally, the Federal Ministry of Education, Science, and Research published the study link and recommended participation on its website.

In Austria, universities stopped providing onsite learning on March 16. Also, as of March 16, the government announced that residents could leave their homes only for work, making necessary purchases, assisting other people, or outdoor exercise alone or in the company of people living in the same household. Beginning on April 6, residents were required to wear face masks in stores and on public transport (Federal Ministry of Health, 2020). During the entire period of data collection, universities ensured continued education by providing distance learning.

#### Study 2: Finland

The sample comprised 1,653 university students (19.6% males, 78.5% females, 1.9% diverse) with a mean age of 28.49 years (*SD* = 8.93, *Mdn* = 25.00, range = 19–69). The students were from the University of Helsinki, Finland. Data were collected from April 29 to June 2, 2020. The link to the online questionnaire was distributed via faculty email lists and the University of Helsinki’s social media channels.

In Finland, universities stopped providing onsite learning on March 18. Universities ensured continued education by providing distance learning. As of May 14, the national government allowed a reopening of higher education institutions. However, it was strongly suggested that the institutions would stay closed the whole semester. The University of Helsinki was closed during the entire period of the data collection.

### Measures

Due to the novelty of the COVID-19 situation, we adapted existing scales or developed new items to suitably address the current circumstances. To ensure content validity of the measures, we revised the items in a first step based on expert judgments from members of our research group. In a next step, the questionnaire was piloted with cognitive interview testing. Finally, the original German questionnaire was translated into Finnish using the translation–back-translation method (Brislin, 1986). To ensure the construct validity of the finally implemented measures, we conducted confirmatory factor analyses (CFAs) and analyzed composite reliability (CR; Raykov, 2009). According to common cutoff criteria for reliability, CR scores above .60, .70, .80, and .90 are deemed marginal, acceptable, good, and excellent, respectively (Hair et al., 2010). All items in the questionnaire were rated on a 5-point Likert-type scale, ranging from 1 (*strongly agree*) to 5 (*strongly disagree*). Participants were instructed to respond to items with respect to the current situation, that is, learning from home due to the COVID-19 pandemic. In order to simplify the interpretation of results, all analyses were conducted with recoded items so that higher values reflected higher agreement with the statements.

*Competence* was measured with three items adapted from the Work-related Basic Need Satisfaction Scale (Van den Broeck et al., 2010). We adapted the work-related items to the university context (sample item: “Currently, I am dealing well with the demands of my studies”). The scale’s CR was .79 for Austria and .90 for Finland.

*Autonomy* was assessed with three newly developed items that addressed the extent to which students felt that they were self-determined in approaching their studies in the current situation (sample item: “Currently, I can perform tasks in the way that best suits me”; CR = .65 and .66 for Austria and Finland, respectively).

*Relatedness* was measured with three items inspired by the Work-Related Basic Need Satisfaction Scale (Van den Broeck et al., 2010) and the German Basic Psychological Need Satisfaction and Frustration Scale (Heissel et al., 2018). In contrast to competence and autonomy, the items targeting relatedness did not refer solely to the university context but also to significant others in general (sample item: “Currently, I feel connected with the people who are important to me”; CR = .75 and .82 for Austria and Finland, respectively).

*Self-regulated learning* in terms of goal setting and planning one’s learning process was assessed with three items, slightly adapted from the short version of the Learning Strategies of University Students questionnaire (Klingsieck, 2018; sample item: “In the current home-learning situation, I plan my course of action”; CR = .78 and .76, for Austria and Finland, respectively).

*Positive emotion* was measured with two items inspired by the Scale of Positive and Negative Experience (Diener et al., 2010; “I feel good,” “I feel confident”) and one item adapted from the optimism subscale of the EPOCH Measure of Adolescent Well-Being (Engagement, Perseverance, Optimism, Connectedness, and Happiness; Kern et al., 2016; “Even if things are difficult right now, I believe that everything will turn out all right”; CR = .85 and .87 for Austria and Finland, respectively).

*Intrinsic learning motivation* was assessed with three items slightly adapted from the Scales for the Measurement of Motivational Regulation for Learning in University Students (A. E. Thomas et al., 2018; sample item: “Currently, I am really enjoying studying and doing work for university”; CR = .92 and .86 for Austria and Finland, respectively).

### Data Analysis

Data were analyzed using SPSS Version 25.0 and Mplus Version 8.4 (Muthén & Muthén, 2017). We conducted CFAs and structural equation models with latent interactions. The proportion of missing values ranged from 0.3% to 4.5% on the item level. To deal with the missing values, the full information maximum likelihood approach was employed. Statistical significance testing was performed at the .05 level. Due to the large sample size, rather than relying on statistical significance, we focused on the effect sizes of the regression parameters when interpreting the results. We followed Cohen’s (1988) recommendations, according to which standardized values of 0.10, 0.30, and 0.50 reflect small, moderate, and large effects.

First, CFAs using robust maximum likelihood estimation were conducted to analyze the construct validity of the scales. Goodness-of-fit was evaluated using χ² test of model fit, the comparative fit index (CFI), and the root mean square error of approximation (RMSEA). Following Hu and Bentler (1999), CFI > .95 and .90 and RMSEA < .06 and .08 represent excellent and adequate model fit, respectively.

Second, we tested for measurement invariance across countries of data collection. CFAs for the three basic needs, self-regulated learning, and the outcomes were set up to investigate the dimensionality of the scales. We tested for measurement invariance (configural invariance, metric invariance, and scalar invariance) across countries by calculating a set of increasingly constrained CFAs. While configural invariance tests whether the same factor structure is valid for each group, metric invariance indicates that participants in both countries attribute the same meaning to the latent constructs. Finally, if the assumption of scalar invariance holds, the meaning of the levels of the underlying items is equal in both groups (van de Schoot et al., 2012). We followed Chen (2007) when evaluating the measurement invariance assumptions. Accordingly, when the sample size is adequate (*N* > 300), declines in CFI > .01 and increases in RMSEA > .015 indicate meaningful model fit changes, making the assumptions of measurement invariance not tenable. For all three models, which were measured on 5-point Likert-type scales, the robust maximum likelihood estimator was used for the CFAs.

Third, we set up two models to test the main effects and the latent interactions in both studies. Model 0 tested the main effects of competence, autonomy, relatedness, and self-regulated learning on positive emotion and intrinsic learning motivation. In Model 1, latent interactions between competence and self-regulated learning as well as between autonomy and self-regulated learning were added, as appropriately specified latent-interaction models include both main-effect variables and the product term (Cohen, 1978; Cronbach, 1987). Additionally, following Maslowsky et al. (2015), we compared the relative fit of Model 0 and Model 1 using a log-likelihood ratio test. A significant log-likelihood ratio test indicates that Model 0 represents a significant loss in fit compared to the more complex Model 1 (Satorra, 2000).

## Results

### Preliminary Analyses

Table 1 provides bivariate latent correlations among all variables as well as descriptive statistics and CRs in both samples.

**Table 1:** Bivariate Latent Correlations, Descriptive Statistics, and Composite Reliabilities for Study 1 and Study 2

Variable/descriptive statistic	1	2	3	4	5	6
1. Competence	—	.57	.37	.42	.69	.53
2. Autonomy	.84	—	.22	.41	.47	.50
3. Relatedness	.31	.27	—	.15	.35	.34
4. Self-regulated learning	.32	.35	.15	—	.33	.43
5. Positive emotion	.64	.51	.23	.16	—	.46
6. Intrinsic learning motivation	.76	.72	.24	.34	.56	—
Study 1: Austria						
No. of items	3	3	3	3	3	3
*M*	3.27	2.93	3.15	3.29	3.68	2.77
*SD*	1.00	0.95	0.95	1.02	0.86	1.14
Range	4.00	4.00	4.00	4.00	4.00	4.00
Composite reliability	.79	.65	.75	.78	.85	.92
Study 2: Finland						
No. of items	3	3	3	3	3	3
*M*	3.62	3.37	3.40	3.39	3.53	3.40
*SD*	1.03	0.82	0.90	0.93	0.85	0.94
Range	4.00	4.00	4.00	4.00	4.00	4.00
Composite reliability	.90	.66	.82	.76	.87	.86

*Note. N_Study1_* = 6,071, *N_Study2_* = 1,653. All scales were 5-point Likert-type scales. Correlations for Study 1 (Austria) are below the diagonal and correlations for Study 2 (Finland) are above the diagonal. All correlation coefficients are statistically significant at *p* < .001.

Due to the high correlation between competence and autonomy in Study 1, we investigated collinearity of the predictors and computed the variance inflation factor (VIF) for the two variables, yielding VIF_comp_ = 3.94 and VIF_auto_ = 3.40. Generally, VIFs higher than 5 are considered to indicate potential difficulties in separating out the independent contribution of the variables concerned (James et al., 2013). Other authors suggest more conservative cutoffs, considering VIFs greater than 2.5 indicative of collinearity (Johnston et al., 2018). If the stricter criterion is applied, the effects of competence and autonomy on the outcomes in Study 1 should be interpreted cautiously, as it cannot be ruled out that the respective slope parameters are over- or underestimated.

### Confirmatory Factor Analyses and Measurement Invariance Testing

The CFAs revealed excellent fit indices for all scales for both Study 1, χ²(120) = 1963.24, *p* < .001, RMSEA = .050, CFI = .959, and Study 2, χ²(120) = 515.09, *p* < .001, RMSEA = .045, CFI = .968. The tests for measurement invariance showed that configural and metric invariance could be established for all variables based on Chen’s (2007) recommendations. As for scalar invariance, considering the declines in CFI greater than .01 in all three models and increases in RMSEA greater than .015 for self-regulated learning and the outcome variables, the scalar invariance assumptions did not hold. Accordingly, the meanings of the levels of the items were not equal in both groups. Therefore, while the same factor structure and the same meanings attributed to the latent constructs can be assumed, factor means should not be compared across the two countries of data collection. Results of the measurement invariance testing are reported in Table 2.

**Table 2:** Measurement Invariance Testing Across Countries for the Confirmatory Factor Analytic Measurement Models for Basic Psychological Needs, Self-Regulated Learning, Learning Motivation, and Positive Emotion

Model	χ^2^	*df*	CFI	ΔCFI	RMSEA	ΔRMSEA	BIC	Model description
Basic psychological needs (competence, autonomy, relatedness)								
	813.273*	48	0.962		0.064		196270.416	Configural invariance
	867.890*	54	0.959	−0.003	0.063	−0.001	196275.910	Metric invariance
	1309.725*	60	0.937	−0.022	0.074	0.011	196718.698	Scalar invariance
Self-regulated learning								
	0.000	0	1.000		0.000		66283.023	Configural invariance
	32.920*	2	0.993		0.064		66302.676	Metric invariance
	292.518*	4	0.933	−0.06	0.138	0.074	66561.219	Scalar invariance
Outcomes (intrinsic learning motivation, positive emotion)								
	209.779*	16	0.991		0.056		109910.784	Configural invariance
	245.671*	20	0.989	−0.002	0.054	−0.002	109909.441	Metric invariance
	561.627*	24	0.975	−0.014	0.076	0.022	110223.000	Scalar invariance

*Note.* Note that the model for self-regulated learning had only three factor indicators and that the configural invariance model was therefore saturated; χ² = chi-square test of model fit; *df* = degrees of freedom; CFI = comparative fit index; ΔCFI = change in CFI compared to the weaker measurement invariance model above; RMSEA = root mean square error of approximation; ΔRMSEA = change in RMSEA compared to the weaker measurement invariance model above; BIC = Bayesian information criterion.

* *p* ≤ .001.

### Main Effects and Latent Interactions

To analyze the main effects of the three basic needs and self-regulated learning on positive emotion and intrinsic learning motivation, we conducted two structural equation models (Model 0_1_ and Model 0_2_) for the two studies. Model estimation for the main effect models revealed that competence positively predicted both outcomes in both studies. Autonomy positively predicted intrinsic learning motivation in both studies, and positive emotion in Finland. Albeit the small effect size, autonomy was identified as a negative predictor for positive emotion in Austria. In this regard, however, it must be noted that competence and autonomy were highly correlated in the Austrian sample. Therefore, the parameter estimates for the unique effects of competence and autonomy on the outcomes might not be reliable. Relatedness positively predicted positive emotion in both studies, and intrinsic learning motivation in Finland only, with small effect sizes, respectively. Self-regulated learning negatively predicted positive emotion with a small effect in Austria, and positively predicted intrinsic learning motivation in both studies (see Table 3 for Austria and Table 4 for Finland). According to the path coefficients, competence was the relatively more important predictor for both outcomes in Austria, and for positive emotion in Finland. In Finland, autonomy exerted the greatest effect on intrinsic learning motivation. However, besides comparing the coefficients on the descriptive level, we tested the differences of the regression slopes for each outcome variable for statistical significance using the MPlus Model Constraint command. In Austria, all regression coefficients, except for those between autonomy and positive emotion and between self-regulated learning and positive emotion, were statistically significantly different from each other. In Finland, all coefficients for positive emotion differed significantly, except for the regression coefficients of autonomy and relatedness. Regarding the effects on intrinsic learning motivation in Finland, there were no statistically significant differences between the regression coefficients of competence and autonomy, of competence and self-regulated-learning, and of autonomy and self-regulated learning.

**Table 3:** Path Coefficients of the Main Effect Model (Model 0) and the Latent-Interaction Model (Model 1) for Study 1

Outcome and predictor	Model 0_1_			Model 1_1_		
	Est. (*SE*)	Std. Est.	*p*	Est. (*SE*)	Std. Est.	*p*
Positive emotion						
Competence	0.57 (0.03)	0.71	<.001	0.58 (0.03)	0.72	<.001
Autonomy	−0.06 (0.03)	−0.08	.047	−0.08 (0.03)	−0.10	.021
Relatedness	0.03 (0.01)	0.04	.011	0.03 (0.01)	0.04	.009
SRL	−0.04 (0.02)	−0.05	.004	−0.04 (0.02)	−0.04	.012
Competence × SRL				−0.06 (0.05)	−0.06	.213
Autonomy × SRL				0.11 (0.05)	0.11	.038
*R*²	.41			.42		
Intrinsic learning motivation						
Competence	0.61 (0.04)	0.51	<.001	0.62 (0.04)	0.52	<.001
Autonomy	0.32 (0.04)	0.27	<.001	0.30 (0.04)	0.25	<.001
Relatedness	0.00 (0.01)	0.00	0.980	0.00 (0.01)	0.00	.924
SRL	0.11 (0.02)	0.08	<.001	0.12 (0.02)	0.09	<.001
Competence × SRL				0.07 (0.06)	0.05	.214
Autonomy × SRL				0.02 (0.06)	0.01	.777
*R*²	.61			.61		
Goodness of fit						
AIC	290942.372			290910.182		
BIC	291405.428			291400.082		

*Note.* SRL = self-regulated learning; Est. = unstandardized parameter estimate; Std. Est. = standardized estimate; AIC = Akaike information criterion; BIC = Bayesian information criterion.

**Table 4:** Path Coefficients of the Main Effect Model (Model 0) and the Latent-Interaction Model (Model 1) for Study 2

Outcome and predictor	Model 0_2_			Model 1_2_		
	Est. (*SE*)	Std. Est.	*p*	Est. (*SE*)	Std. Est.	*p*
Positive emotion						
Competence	0.45 (0.03)	0.59	<.001	0.47 (0.03)	0.60	<.001
Autonomy	0.10 (0.03)	0.11	.003	0.09 (0.03)	0.10	.005
Relatedness	0.07 (0.02)	0.10	<.001	0.07 (0.02)	0.10	<.001
SRL	0.02 (0.03)	0.03	.409	0.02 (0.03)	0.02	.434
Competence × SRL				0.08 (0.04)	0.08	.047
Autonomy × SRL				−0.06 (0.05)	−0.05	.240
*R*²	.50			.51		
Intrinsic learning motivation						
Competence	0.23 (0.04)	0.25	<.001	0.21 (0.04)	0.23	<.001
Autonomy	0.27 (0.04)	0.24	<.001	0.27 (0.04)	0.24	<.001
Relatedness	0.14 (0.02)	0.16	<.001	0.14 (0.02)	0.16	<.001
SRL	0.24 (0.04)	0.21	<.001	0.25 (0.04)	0.21	<.001
Competence × SRL				−0.06 (0.05)	−0.05	.266
Autonomy × SRL				−0.02 (0.06)	0.01	.753
*R*²	.39			.39		
Goodness of fit						
AIC	73915.633			73907.526		
BIC	74288.947			74302.481		

*Note.* SRL = self-regulated learning; Est. = unstandardized parameter estimate; Std. Est. = standardized estimate; AIC = Akaike information criterion; BIC = Bayesian information criterion.

**Figure 1.**
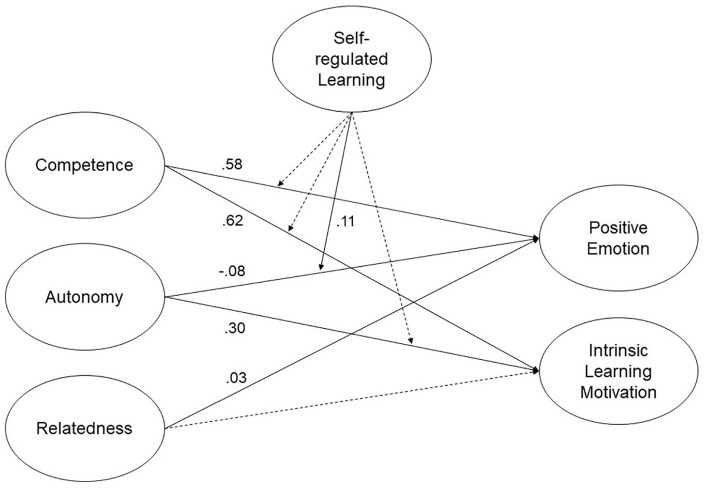
Structural equation model predicting positive emotion and intrinsic learning motivation (Study 1: Model 1_1_). *Note*. This structural equation model predicts positive emotion and learning motivation from basic psychological needs, with moderating effects of self-regulated learning. Statistics are standardized regression coefficients. Dotted lines represent nonsignificant relations.

To investigate moderation effects of self-regulated learning on the outcomes, latent interactions (competence × self-regulated learning and autonomy × self-regulated learning) were added to Model 0 for both studies, resulting in Model 1_1_ and Model 1_2_. In Austria, a statistically significant positive latent interaction emerged between autonomy and self-regulated learning for positive emotion, b* = 0.11, *SE* = 0.05, *p* = .038 (see Model 1_1_ in Table 3). Therefore, the effect of autonomy varied with the level of self-regulated learning for positive emotion. In other words, the effect of autonomy on positive emotion increased, as the moderator increased, and vice versa. The relative fit of Model 1_1_ versus Model 0_1_ was determined via a log-likelihood ratio test, yielding a significant log-likelihood difference of *D*(4) = 472.778, *p* < .001. This result indicates that the null model represents a significant loss in fit relative to Model 1_1_. Model 1_1_ should therefore be kept for Study 1 (see Figure 1).

**Figure 2.**
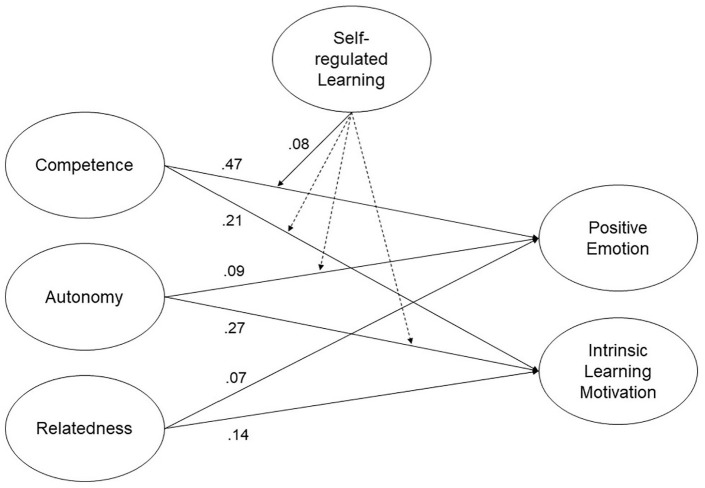
Structural equation model predicting positive emotion and intrinsic learning motivation (Study 2: Model 1_2_). *Note*. This structural equation model predicts positive emotion and learning motivation from basic psychological needs, with moderating effects of self-regulated learning. Statistics are standardized regression coefficients. Dotted lines represent nonsignificant relations.

In Finland, a statistically significant positive latent interaction emerged between competence and self-regulated learning for positive emotion, b* = 0.08, *SE* = 0.04, *p* = .047 (see Model 1_2_ in Table 4). Therefore, the effect of competence on positive emotion increased, as the level of self-regulated learning increased, and vice versa. The log-likelihood ratio test was significant with *D*(4) = 16.108, *p* < .005, indicating a better relative fit for Model 1_2_. Model 1_2_ is therefore kept for Study 2 (see Figure 2).

## Discussion

The present research aimed at identifying resources that relate to university students’ psychological well-being in the challenging period of the COVID-19 pandemic. Following SDT (Deci & Ryan, 2000), we examined to what extent basic psychological need satisfaction related to university students’ psychological well-being when involuntarily learning from home during the COVID-19 pandemic. Additionally, we considered the role of self-regulated learning as a moderator. To examine whether evidence is consistent across countries, we took a multistudy approach and collected data in Austria (Study 1) and Finland (Study 2).

Based on SDT, we expected that all three basic needs, that is, experienced competence, autonomy, and relatedness, predicted psychological well-being in terms of positive emotion and intrinsic learning motivation. In contrast to a broad body of research clearly pointing to the assumed associations (e.g., Reinboth & Duda, 2006; Riggenbach et al., 2019; Van den Broeck et al., 2016), our results only revealed competence to predict positive emotion with a large effect in Austria, and a moderate effect in Finland. In Austria, relatedness was a further positive predictor and autonomy was a negative predictor of positive emotion. In Finland, both autonomy and relatedness were further positive predictors of positive emotion. However, the minor to small effect sizes of autonomy and relatedness in both studies do not allow to conclude on the practical relevance of the identified coefficients. Moreover, due to potential collinearity of competence and autonomy in the Austrian sample, it must be assumed that the unique effect of autonomy on positive emotion was underestimated. This is also underlined by the high bivariate correlation between autonomy and positive emotion in Austria (Hypothesis 1a). Regarding associations of the basic needs and intrinsic learning motivation, competence and autonomy were positive predictors with a large (competence) and a moderate (autonomy) effect on the outcome in Austria. However, the possible collinearity of these two predictors must also be considered here. Accordingly, the unique contribution of competence and autonomy might have been incorrectly estimated for the Austrian sample. In Finland, all three basic needs positively predicted intrinsic learning motivation with small to moderate effect sizes (Hypothesis 1b).

The assumed moderation effects of self-regulated learning on the relationship between autonomy and competence and the outcomes were inconsistent. While we found a statistically significant effect for the interaction between self-regulated learning and autonomy on positive emotion in Austria, we identified a statistically significant effect for the interaction between self-regulated learning and competence on positive emotion in Finland (Hypotheses 2a and 2b). For the moderation effect of self-regulated learning on the relationships between competence and intrinsic learning motivation, and between autonomy and intrinsic learning motivation, we found no significant effects (Hypotheses 2c and 2d). Although the interactions were not consistently identified, it should be noted that the main effects of self-regulated learning on intrinsic learning motivation were significant with small to moderate effect sizes in both studies. This speaks in favor of the relevance of self-regulated learning for enabling intrinsic learning motivation.

On the whole, the results of the analyses were broadly consistent across both Study 1 and Study 2, providing convergent evidence across countries. This applies in particular to the identified high relevance of experienced competence for positive emotion and the relevance of autonomy and self-regulated learning for intrinsic learning motivation. Both studies further indicated an only minor relevance of relatedness for positive emotion. This unexpected finding could be due to the fact that in the time of the pandemic, social contacts play a different role than under usual circumstances, and that the directive to reduce face-to-face contacts could have resulted in social contacts having a different connotation overall: to withdraw and refrain from social contact in order to be protected from the virus and feel safe. In this respect, the term *cocooning* had a revival, referring to staying inside one’s private home, shielded from perceived danger, instead of going out (Merriam-Webster, 2020; Oxford English Dictionary, 2020). With respect to the COVID-19 pandemic, cocooning has been referred to as self-isolation of the elderly and risk groups (Duque et al., 2020) but has also been positively connotated as a lifestyle trend among young people that is about peace, protection, coziness, and control (see Popcorn, 1992).

### Implications for Higher Education in Times of COVID-19

Both studies identified a high relevance of experienced competence for positive emotion and the relevance of autonomy and self-regulated learning for intrinsic learning motivation. In addition, there are indications that, to a certain extent, relatedness has a positive influence on intrinsic learning motivation. All three basic needs as well as self-regulated learning can be specifically promoted in the university context through distance learning.

Based on the identified high relevance of experienced competence, distance education in times of COVID-19 is required to explicitly enable students to experience successes. This can be achieved through individualized and at the same time autonomy-supportive learning opportunities, challenging students based on their individual strengths and weaknesses. Experiencing success can be further promoted by setting intermediate goals. In addition, there should be enough room for individual feedback (see, e.g., Lawn et al., 2017; Oliveira et al., 2018; Paechter & Maier, 2010). To foster self-regulated learning, which proved to be a positive predictor for intrinsic learning motivation, universities should instruct students to structure and plan their learning consciously. The necessity to explicitly convey strategies for self-regulated learning is underlined by studies, according to which students know about them in theory but in many cases do not use self-regulated learning strategies in everyday life and perceive them as tedious and unnecessary (e.g., Foerst et al., 2017). Promoting the explicit use of self-regulated learning strategies is a relevant short-term objective in the current distance learning situation, but it also bears the potential to equip students for lifelong learning in general (Lüftenegger et al., 2012).

Finally, when it comes to promoting relatedness and identification with the university in the current situation, digital learning platforms can be used to enable online group work at a physical distance. To foster the feeling of learning together as a group, synchronous learning units (e.g., video group calls) could be used to reflect on learning processes, successes, as well as struggles and to promote cohesion within the group. For further strategies to promote the development of an online community, see, for example Rovai (2007).

### Limitations and Future Directions

We consider the high explanatory power of our models, with proportions of explained variance ranging from 39% to 61%, and the large sample size as substantial strengths of our research. Moreover, we followed a multistudy approach and tested our assumptions in samples from two different countries. The results are further supported by the identified configural and metric measurement invariance, indicating that the same factor structure and the same meanings attributed to the latent constructs can be assumed across both countries of data collection.

Despite these noteworthy strengths, the present research is limited in some respects. First, the results rely on self-reports. While this is the usual practice for most of the examined constructs, there are concerns about the validity of self-report tools for assessing self-regulated learning (e.g., Winne et al., 2002). Second, data were collected online. This led to a self-selection of our sample and as a consequence to an overrepresentation of females. In addition, the sample sizes of Study 1 and Study 2 differ greatly, leading to inequivalent statistical power to detect main and moderating effects. A further limitation relates to the cross-sectional design of our research, limiting the possibility for causal inferences. Finally, due to the novelty of the COVID-19 situation, some of our measures were newly developed for this study. Because of the urge to quickly start collecting data to generate information about the sudden situation, it was not possible to carry out a comprehensive validation study of the instruments. Nevertheless, we can account for the validity of our instruments in several ways, including cognitive interview testing, CFAs, CR, and measurement invariance testing.

Considering these limitations, we recommend that follow-up studies incorporate further informants (e.g., teacher ratings, observations) and other methods of data collection (e.g., experience sampling, in-depth qualitative methods) to obtain a more comprehensive picture. This especially relates to the role of self-regulated learning and to the further investigation of the surprisingly low association between relatedness and positive emotion. Future research should also consider different approaches of sample recruitment to ensure a better representation of the population. Particularly, longitudinal studies should be carried out to further substantiate the evidence for the large effects found. With regard to the delivery of distance education, it should be considered to evaluate concrete design options such as modality, pacing, instructional practices, role of assessments, and feedback (see Means et al., 2014) and to investigate the extent to which they are suitable to enable experience of competence and to support self-regulated learning.

## Conclusion

The present research highlights the relevance of perceived competence, autonomy and self-regulated learning for university students’ well-being in times of unplanned and involuntary remote studying. The results also indicate a potential relevance of relatedness for intrinsic learning motivation. The requirement for higher education institutions is to explicitly promote these identified dimensions. To take advantage of the potential, distance learning should be designed in a way that maximizes the strengths and constrains the weaknesses of distance education.
